# Patterns of Polysubstance Use Among Non-Hispanic White and American Indian/Alaska Native Adolescents: An Exploratory Analysis

**DOI:** 10.5888/pcd16.180108

**Published:** 2019-04-04

**Authors:** Lee Kiedrowski, Arielle Selya

**Affiliations:** 1 University of North Dakota School of Medicine & Health Sciences, Grand Forks, North Dakota; 2 Department of Population Health, University of North Dakota School of Medicine and Health Sciences, Grand Forks, North Dakota; 3 Behavioral Sciences Group, Sanford Research, Sioux Falls, South Dakota; 4 Department of Pediatrics, University of South Dakota Sanford School of Medicine, Sioux Falls, South Dakota

## Abstract

**Introduction:**

The prevalence of polysubstance use is well described, but less is known about correlates and patterns of polysubstance use. Previous research characterized latent subgroups of substance users by the type substance used. Racial disparities in the prevalence of polysubstance use exist, particularly for American Indian/Alaska Native (AI/AN) populations. The objective of our study was to describe differences in patterns of polysubstance use between non-Hispanic white and AI/AN adolescents.

**Methods:**

We obtained data from the 2013 Youth Risk Behavior Survey (YRBS). We analyzed substance use patterns (cigarettes, alcohol, marijuana, cocaine, inhalants, heroin, methamphetamines, ecstasy, steroids, and injected drugs) separately among 375 AI/AN and 15,633 non-Hispanic white adolescents. We calculated pairwise correlations. Exploratory factor analysis identified latent factors of polysubstance use patterns.

**Results:**

The use of all substances by AI/AN adolescents was the same or higher than use by non-Hispanic white adolescents, particularly for cocaine, heroin, and steroids. We found strong correlations between use of heroin and injected drugs and between use of cocaine and ecstasy among both populations. We found a latent factor for cigarettes, alcohol, and marijuana and another factor for broad polysubstance use among both populations. We found a factor for steroids and injected drugs among AI/AN adolescents, a factor for cocaine and ecstasy among non-Hispanic white adolescents, and a unique factor for methamphetamines.

**Conclusion:**

Differences in substance use patterns exist between AI/AN and non-Hispanic white adolescents, particularly for illegal drug use. If validated in future research, information on these differences could be used to inform tailored intervention programs aimed at preventing substance use.

**Summary**

**What is already known on this topic?**

Polysubstance use among adolescents in the United States is rising, and rates of substance use differ by race/ethnicity. Typically, substance use among non-Hispanic white adolescents is less prevalent than among American Indian/Alaska Native adolescents.

**What is added by this report?**

American Indian/Alaska Native adolescents tended to use steroids and injected drugs together, whereas non-Hispanic white adolescents used cocaine and ecstasy together. Both groups used tobacco, marijuana, and alcohol together and a cluster of other illicit substances together.

**What are the implications for public health practice?**

These findings can be used in the clinical setting to screen for and prevent substance and polysubstance use among adolescents.

## Introduction

Substance use is well understood to have negative, life-long consequences. Particularly concerning is the use of substances among adolescents, because initiation often occurs during these formative years, thus increasing the likelihood of addiction and continued use (1). In 2013, 7.8% of adolescents used tobacco, 22.7% drank alcohol, and 8.8% used illegal drugs (2,3).

Prevalence of substance use is well known for individual substances at the general population level; however, substance use differs widely across subgroups of the population. For example, with the exception of alcohol, substance use is more prevalent among American Indian/Alaska Native (AI/AN) populations than among white populations (3). In addition to racial disparities in the prevalence of substance use, racial differences in risk factors and correlates of risky behaviors exist (4–6).

Even after accounting for demographic characteristics, adolescent populations have distinct heterogeneous subgroups (ie, latent classes) that differ in their patterns of polysubstance use (7–9). Common subgroups include nonusers or infrequent users, broad polysubstance users, and users of 1 or more substances (7), such as alcohol (7,8,10) or dual use of marijuana and cigarettes (8). These subgroups differ in their environmental-level and/or individual-level risk factors for substance use (7).

Considering that racial differences exist among these social and environmental risk factors for substance use, it is plausible that common patterns of substance use could fundamentally differ by racial or ethnic group. Little is known about whether such differences in patterns of substance use exist between AI/AN and non-Hispanic white adolescents. If differences do exist, then that information could be used to identify adolescents at higher risk for polysubstance use. Effective treatment plans and education could then be designed and implemented to prevent additional substance use. Our study used data from the 2013 Youth Risk Behavior Survey (YRBS) to examine potential differences in correlations and patterns of substance use between AI/AN and non-Hispanic white adolescents.

## Methods

We used publicly available data from the 2013 YRBS (the most recent data available at the beginning of this research, in March 2015). The YRBS is an observational, cross-sectional survey designed in 1990 by the Centers for Disease Control and Prevention to monitor the leading risk behaviors of US adolescents and young adults that contribute to death, disability, and social problems (11).

We limited our analysis to 2 self-reported race/ethnicities: AI/AN (n = 1,096) and non-Hispanic white (n = 45,187). We found a substantial amount of missing data on substance use variables, which was handled with listwise deletion, resulting in a final sample size of 375 AI/AN adolescents and 15,633 non-Hispanic white adolescents. We used listwise deletion because research shows that it is preferable to other methods (eg, imputation) in pattern-based analyses such as ours (12).

The University of North Dakota Institutional Review Board approved this study. All analyses were conducted at the University of North Dakota, Grand Forks, North Dakota, from March 2015 through February 2018.

### Measures

We analyzed survey data on the following substances: tobacco (cigarettes), alcohol, marijuana, cocaine, inhalants, heroin, methamphetamines, ecstasy, steroids, and injected drugs.

Cigarette and alcohol use were each assessed by the self-reported number of days of the past 30 days that each substance was used. Original responses were given as 0 days, 1 or 2 days, 3 to 5 days, 6 to 9 days, 10 to 19 days, 20 to 29 days, or all 30 days. We used the midpoint value of each category for the analyses of the responses.

Marijuana was assessed by the self-reported number of lifetime uses of marijuana. Original responses were given as 0 times, 1 or 2 times, 3 to 9 times, 10 to 19 times, 20 to 39 times, 40 to 99 times, or 100 or more times. We used the midpoint value of each category for the analyses of the responses, and values were top coded at 100.

Cocaine (including “powder, crack, or freebase”), inhalants (including “glue, aerosol spray cans, paints, or sprays to get high”), heroin (including “smack, junk, or China white”), methamphetamines (including “speed, crystal, crank, or ice”), ecstasy (including “MDMA”), and steroids (“steroid pills or shots without a doctor’s prescription”) were assessed by the self-reported number of days in the participant’s lifetime that each substance was used. Original responses were given as 0 times, 1 or 2 times, 3 to 9 times, 10 to 19 times, 20 to 39 times, or 40 or more times. We used the midpoint value of each category for the analyses of the responses, and values were top coded at 40.

The use of injected drugs (“used a needle to inject any illegal drug”) was assessed by the self-reported number of days in the participant’s lifetime that the substance was used. Original responses were given as 0 times, 1 time, or 2 or more times. These values were analyzed numerically and were top coded at 2.

### Analysis

The YRBS has a complex, multistage sampling process, and survey weights are available to account for this sampling design in standard analyses (eg, regressions). However, we intentionally did not account for survey weights because 1) our study was exploratory and 2) standard and automatic procedures for incorporating survey weights are lacking for the type of analysis we conducted. In other words, our focus was to examine broad, qualitative differences in *patterns* of polysubstance use across AI/AN and non-Hispanic white samples rather than to produce population-level parameter estimates.

To quantify the pairwise association between each possible pair of substances, we calculated Spearman ρ, a nonparametric correlation statistic; we used a nonparametric test because the data were not normally distributed. We adjusted for multiple comparisons of the Spearman ρ test by using the Bonferroni correction. We then visualized all pairwise correlations simultaneously by creating a heatmap.

Next, we performed an exploratory factor analysis on each group to identify latent factors based on substance use patterns among each group. Exploratory factor analysis tests the hypothesis that each group (non-Hispanic white and AI/AN) contains subgroups with fundamentally different substance use patterns. Preliminary analyses based on scree plots (not shown) suggested that the optimal number of factors was 2 to 5 factors for the AI/AN group and 4 or 5 factors for the non-Hispanic white group. We selected the final number of factors on the basis of interpretability, model fit, and simplicity of structure (ie, single-loading of variables onto factors with a threshold of 0.3). We used oblique rotation to allow correlation between the latent factors and ordinary least squares factoring to find the minimum residual solution while accounting for nonnormality among variables. We used the Tucker-Lewis Index and the root-mean-square error of approximation to evaluate the goodness-of-fit for the exploratory factor analysis. A Tucker-Lewis Index of 0.9 or greater is considered a good fit (13) as is a root-mean-square error of approximation of 0.05 or less (14).

We conducted all analyses and plots by using R version 3.1.3 (R Foundation for Statistical Computing) run separately for AI/AN and non-Hispanic white samples.

## Results

AI/AN adolescents had a higher rate than non-Hispanic white adolescents of past 30-day use of tobacco and alcohol and lifetime use of marijuana, cocaine, inhalants, heroin, methamphetamines, ecstasy, steroids, and injected drugs (Table 1). Among users of each substance, the median frequency of use was the same or higher among AI/AN adolescents, with large differences found in use of cocaine (14.5 vs 6.0 lifetime uses), heroin (22.0 vs 1.5 lifetime uses), and steroids (29.5 vs 6.0 lifetime uses).

**Table 1:** Descriptive Statistics of Age, Sex, and Frequency of Substance Use Among American Indian/Alaska Native and Non-Hispanic White Adolescents, 2013 Youth Risk Behavior Survey^a^

Characteristic	American Indian/Alaska Native (n = 1,096)	Non-Hispanic White (n = 45,187)
**Age, y**	16.0 (15.0–17.0)	16.0 (15.0–17.0)
**Male sex**	617 (56.6)	23,517 (52.1)
**Use of substance**		
Cigarettes		
Past 30-day use	388 (38.2)	14,782 (33.7)
Frequency among users	14.5 (4.0–30.0)	14.5 (4.0–30.0)
Alcohol		
Past 30-day use	534 (54.2)	22,632 (51.8)
Frequency among users	4.0 (1.5–7.5)	4.0 (1.5–7.5)
Marijuana		
Lifetime use	575 (54.8)	17,952 (40.1)
Frequency among users	29.5 (6.0–100.0)	14.5 (6.0–69.5)
Cocaine		
Lifetime use	161 (15.2)	3,620 (8.1)
Frequency among users	14.5 (1.5–40)	6.0 (1.5–14.5)
Inhalants		
Lifetime use	169 (21.1)	5,438 (16.5)
Frequency among users	6.0 (1.5–29.5)	6.0 (1.5–6.0)
Heroin		
Lifetime use	32 (5.5)	625 (2.7)
Frequency among users	22.0 (6.0–40.0)	1.5 (1.5–29.5)
Methamphetamine		
Lifetime use	90 (15.3)	2,102 (9.1)
Frequency among users	6.0 (1.5–29.5)	6.0 (1.5–14.5)
Ecstasy		
Lifetime use	63 (13.6)	1,713 (10.1)
Frequency among users	1.5 (1.5–14.5)	1.5 (1.5–6.0)
Steroids		
Lifetime use	96 (8.9)	1,809 (4.0)
Frequency among users	29.5 (4.9–40.0)	6.0 (1.5–14.5)
Injected drugs		
Lifetime use	43 (5.3)	721 (2.2)
Frequency among users	2.0 (1.0–2.0)	2.0 (1.0–2.0)

a Data source: Centers for Disease Control and Prevention (11). Frequency for cigarette and alcohol use was defined as the number of days used in the past 30 days. For all other substances, frequency refers to the number of days used in lifetime. Continuous variables are summarized as median (interquartile range). Categorical variables are summarized as number (percentage).

Pairwise correlations showed strong similarities across groups in which pairs of substances were correlated with each other and, in general, the correlations were slightly stronger among AI/AN adolescents than among non-Hispanic white adolescents (Figure). The top 3 pairwise correlations for AI/AN adolescents were heroin with injected drugs (ρ = 0.669, *P* < .001), cocaine with methamphetamines (ρ = 0.661, *P* < .001), and steroids with injected drugs (ρ = 0.599, *P* < .001). The top 3 pairwise correlations for non-Hispanic white adolescents were cocaine with methamphetamines (ρ = 0.614, *P* < .001), heroin with injected drugs (ρ = 0.608, *P* < .001), and cigarettes with marijuana (ρ = 0.585, *P* < .001). Cocaine with methamphetamine and heroin with injected drugs were in the top 3 most strongly correlated pairs among both AI/AN and non-Hispanic white adolescents.

**Figure:**
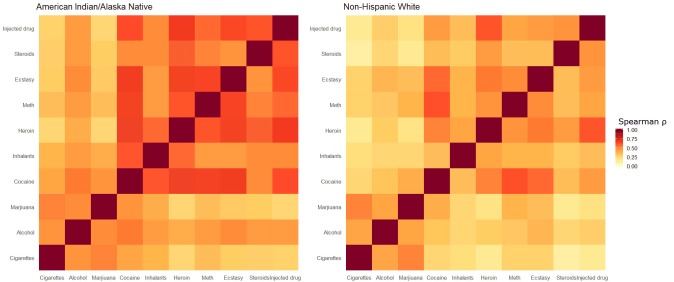
Pairwise correlations (Spearman ρ) among American Indian/Alaska Native and non-Hispanic white adolescents, Youth Risk Behavior Survey, 2013. Each pair of drugs is represented as a square. Abbreviation: Meth, methamphetamine.

In the exploratory factor analyses, a 3-factor solution was chosen for the AI/AN group, and a 4-factor solution was chosen for the non-Hispanic white group (Table 2). Both models showed good fit (Tucker-Lewis Index = 0.91, root-mean-square error of approximation = 0.008 for AI/AN adolescents; Tucker-Lewis Index = 0.99, root-mean-square error of approximation = 0.001 for non-Hispanic white adolescents). Among AI/AN adolescents, one of the latent factors was associated with use of cocaine, ecstasy, heroin, methamphetamines, and inhalants; the second latent factor was associated with use of injected drugs and steroids; and the third latent factor was associated with use of cigarettes, marijuana, and alcohol. Among non-Hispanic white adolescents, one of the latent factors was associated with use of heroin, injected drugs, steroids, and inhalants; the second latent factor was associated with use of cigarettes, marijuana, and alcohol; the third latent factor was associated with use of methamphetamines; and the fourth latent factor was associated with use of ecstasy and cocaine.

**Table 2:** Exploratory Factor Analysis of Patterns of Polysubstance Use Among American Indian/Alaska Native and Non-Hispanic White Adolescents, 2013 Youth Risk Behavior Survey^a^

Substance	American Indian/Alaska Native			Non-Hispanic White			
	Factor 1	Factor 2	Factor 3	Factor 1	Factor 2	Factor 3	Factor 4
Cigarettes	—	—	0.807	—	—	0.748	—
Alcohol	—	—	0.410	—	—	0.512	—
Marijuana	—	—	0.566	—	—	0.730	—
Cocaine	—	0.816	—	—	—	—	0.629
Inhalants	—	0.561	—	—	0.334	—	—
Heroin	—	0.697	—	—	0.743	—	—
Methamphetamine	—	0.641	—	0.980	—	—	—
Ecstasy	—	0.740	—	—	—	—	0.681
Steroids	0.405	—	—	—	0.592	—	—
Injected drugs	0.981	—	—	—	0.729	—	—

a Data source: Centers for Disease Control and Prevention (11). The loading of each variable of substance use onto each factor is shown; blank cells indicate that the loading was below the threshold of 0.3.

These results identified both similarities and differences among AI/AN and non-Hispanic white adolescents. Both groups had a distinct latent factor for cigarettes, alcohol, and marijuana. Other commonalities included a link between 1) heroin and inhalants, 2) steroids and injected drugs, and 3) cocaine and ecstasy. One prominent difference was that methamphetamine was its own independent variable among non-Hispanic white adolescents, but it was linked with other substances among AI/AN adolescents. Additionally, steroids and injected drugs comprised their own latent factor among AI/AN adolescents, whereas cocaine and ecstasy comprised their own latent factor among non-Hispanic white adolescents.

## Discussion

We conducted an exploratory analysis of differences in substance use patterns among AI/AN and non-Hispanic white adolescents in the United States. Pairwise correlations overall showed similar patterns of substance use, particularly for heroin with injected drugs and cocaine with methamphetamines, although the strength of correlations was generally higher among AI/AN adolescents. Exploratory factor analysis identified a common factor for cigarettes, alcohol, and marijuana across both groups, but distinct factors also emerged in each group. Among AI/AN adolescents, injected drugs and steroids comprised a latent factor, whereas among non-Hispanic white adolescents, cocaine and ecstasy comprised a separate latent factor, and methamphetamine alone loaded onto its own factor.

Our study parallels well-known findings about substance use disparities across racial/ethnic groups in adults (15–22). It adds to this body of knowledge by describing differences among adolescents, in particular showing a higher prevalence of substance use among AI/AN adolescents than among non-Hispanic white adolescents. The primary focus of our study, however, extends beyond the prevalence of substance use by examining differences in correlations and patterns of polysubstance use between AI/AN and non-Hispanic white adolescents, about which little is known. Our findings that AI/AN and non-Hispanic white adolescents have different patterns of substance use is consistent in a broader sense with previous findings that risk factors for risky behavior differ between these 2 populations (4–6).

One pattern of substance use common among both AI/AN and non-Hispanic white adolescents was the combination of cocaine and ecstasy; these were strongly associated according to both the correlation analysis and the exploratory factor analysis. Other remarkable correlations in both populations were cigarettes with marijuana and alcohol. Both the heatmap and the factor analysis indicated that if an adolescent used cocaine, he or she would be more likely than not to have also used ecstasy, heroin, methamphetamines, or inhalants. Likewise, if an adolescent used cigarettes, he or she would be more likely than not to have used marijuana and alcohol. Our findings are broadly consistent with previous research on latent classes of substance use (7) in that a pattern emerged for “lighter” substance use (cigarettes, alcohol, and marijuana) and other patterns emerged for “heavier” substance use (substances that are illegal at any age).

We found notable differences between AI/AN and non-Hispanic white adolescents in illegal substance use. In particular, nonmedical steroid use and injected drugs were associated with both AI/AN and non-Hispanic white adolescents, but this pairing comprised its own unique factor among AI/AN adolescents. This pairing indicates that AI/AN adolescents who illicitly use steroids and injected drugs are a distinct subpopulation of AI/AN adolescents. A link between steroid use and injected drugs was reported previously (23), but to our knowledge, this link has not been reported among AI/AN adolescents. Distinct subpopulations of non-Hispanic white adolescents were uniquely characterized by methamphetamine use, indicating that non-Hispanic white methamphetamine users are a distinct subpopulation of non-Hispanic white adolescents. We found this unique characterization despite the overall lower prevalence of methamphetamine use among non-Hispanic white adolescents than among AI/AN adolescents. Similarly, cocaine and ecstasy use characterized another subpopulation of non-Hispanic white adolescents.

Future research should focus on replicating these patterns in other independent samples, examining temporality and possible causality and further refining the exact nature (eg, which drugs are injected and what type of steroids are being used) and timing (eg, whether cocaine and ecstasy are being taken together in a single episode) of these patterns. Validation of racial/ethnic differences in polysubstance use patterns would motivate the development of tailored interventions for subgroups to prevent or reduce the prevalence of polysubstance use.

Strengths of our study include the novel use of exploratory methods to assess polysubstance abuse among AI/AN and non-Hispanic white adolescents. Additionally, our study extends previous research on racial/ethnic disparities in the prevalence of use to examine patterns of polysubstance use. Finally, our study used a large, national data set, which increases the generalizability of the findings to the larger population of high school–aged students in the United States.

Our study had several limitations. First, it used cross-sectional data, which cannot be used to assess causality or rule out residual confounding. Second, because YRBS data are self-reported, they are subject to social desirability and recall biases. Third, the use of cigarettes and alcohol were measured in “use within the past 30 days,” while all other substances were measured in “lifetime use.” This difference results in imperfect direct comparisons between substances. Fourth, the YRBSS has a large degree of missing data on substance use, and these data may not be missing at random. For example, because the sample is limited to students who were at school on the day of the survey, truancy could have affected the findings, especially since truancy is known to be associated with substance use. The patterns of missing data may have affected our findings in unknown ways, and thus the findings should be considered exploratory only. Fifth, it was not possible to examine national YRBS public use data by state or region, and because AI/AN subpopulations can differ by state or region in important ways, further research is needed to explore these differences. Sixth, factor analysis should be considered an exploratory method, and the results may be sensitive to the particular sample or to the specific method of measuring the latent factors (eg, the wording of the questions). Additionally, factor analysis is somewhat subjective, particularly in the way factors are interpreted and the role of interpretability in selecting the number of factors. Finally, several of the substance use items, such as steroids, inhalants, and injected drugs, were broadly defined, and detailed information on the exact substance was not available.

The practicality of our study is noteworthy. The data show strong pairwise correlations between common substances of abuse and classify AI/AN and non-Hispanic white adolescents into well-structured latent factors, indicating that distinct patterns of polysubstance use exist in each subpopulation. A health care professional could use this information to inquire about substance use if a substance is known to be used and subsequently educate susceptible adolescents about substance use habits with the goal of preventing polysubstance abuse. Public health interventions and policy makers can also use these data to formulate strategies for reaching certain racial/ethnic groups to decrease the prevalence of substance abuse with more efficiency and effectiveness.
